# Supplementary material for: Inactivation of PTEN and ZFHX3 in Mammary Epithelial Cells Alters Patterns of Collective Cell Migration
Source: Int J Mol Sci. 2022 Dec 24;24(1):313. doi: 10.3390/ijms24010313 (PMC9820126; doi:10.3390/ijms24010313)
Supplement: Supplementary file 1 [file ijms-24-00313-s001.zip › Supplementary Materials.pdf]

Supplementary Materials

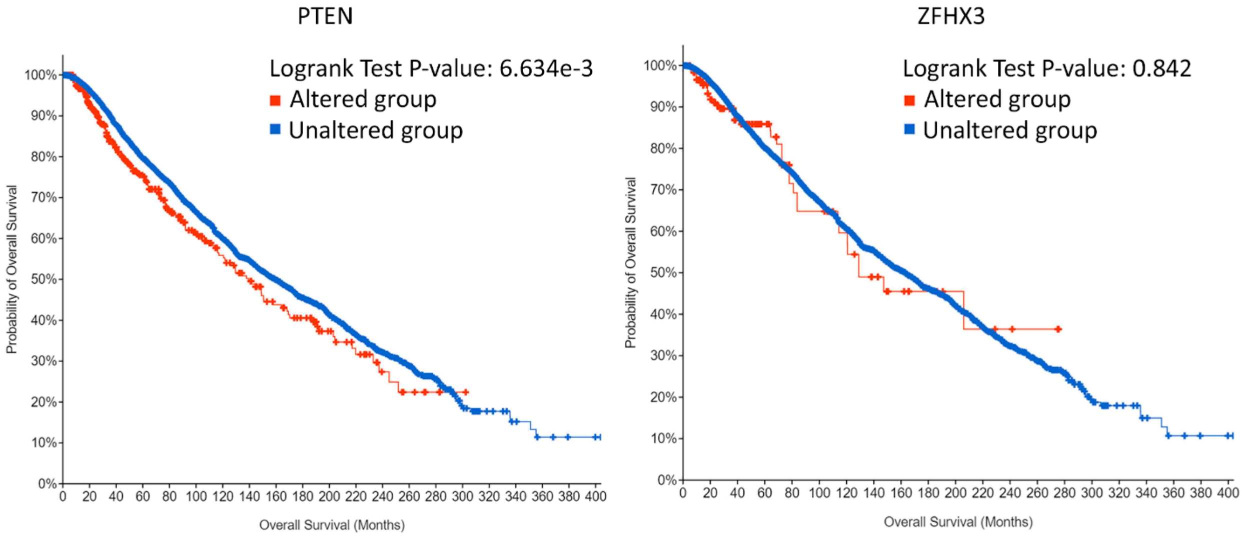

	Number of Cases, Total	Number of Events	Median Months Overall (95% CI)
Altered group	629	179	139.40 (120.57 - 168.30)
Unaltered group	7028	1917	159.20 (150.60 - 168.97)

	Number of Cases, Total	Number of Events	Median Months Overall (95% CI)
Altered group	192	39	128.98 (114.40 - NA)
Unaltered group	7075	1867	163.17 (152.33 - 172.00)

Figure S1. Correlation between PTEN and ZFHX3 mutation status and overall survival in breast cancer patients. Survival was calculated using the cBioPortal tool.

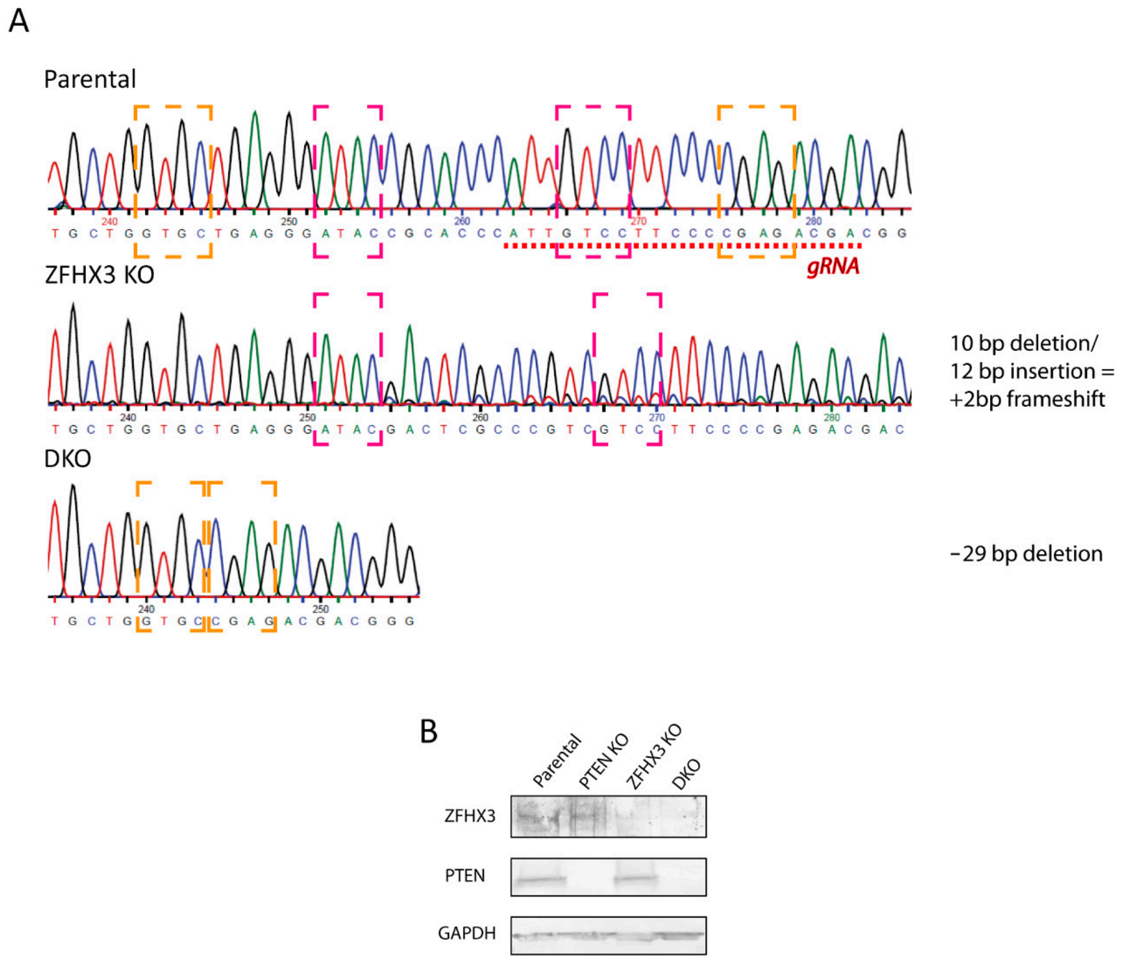

Figure S2. (**A**) *ZFHX3* gene alterations in the single and double KO cell lines studied. The two alterations were observed in genomic PCR products indicating that they were affecting both alleles of the respective cell lines. Both genomic alterations induced a frameshift that generates the appearance of premature stop codons. (**B**) Western blot of parental and gene-edited cell lines for verification of knockouts efficacy.

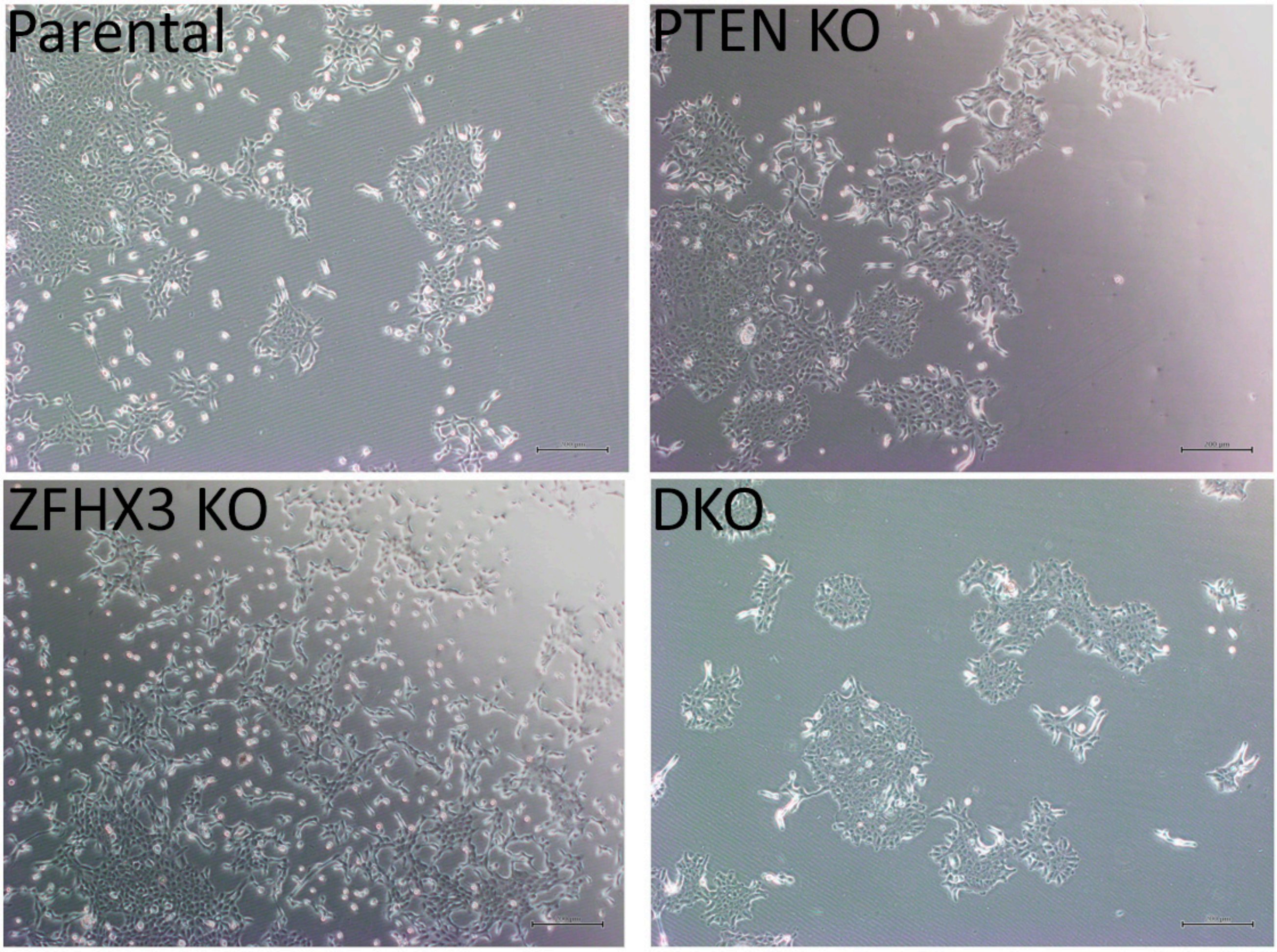

Figure S3. Phase contrast view of the gene-edited MCF10A cell lines, in full medium (5% of horse serum with EGF (epidermal growth factor). Epithelial islets co-exist with isolated cells. PTEN KO cells exhibits small islets and rare isolated cells. On the contrary, in ZFHX3 KO cultures, more isolated cells are present. Scale bars 200 µm.

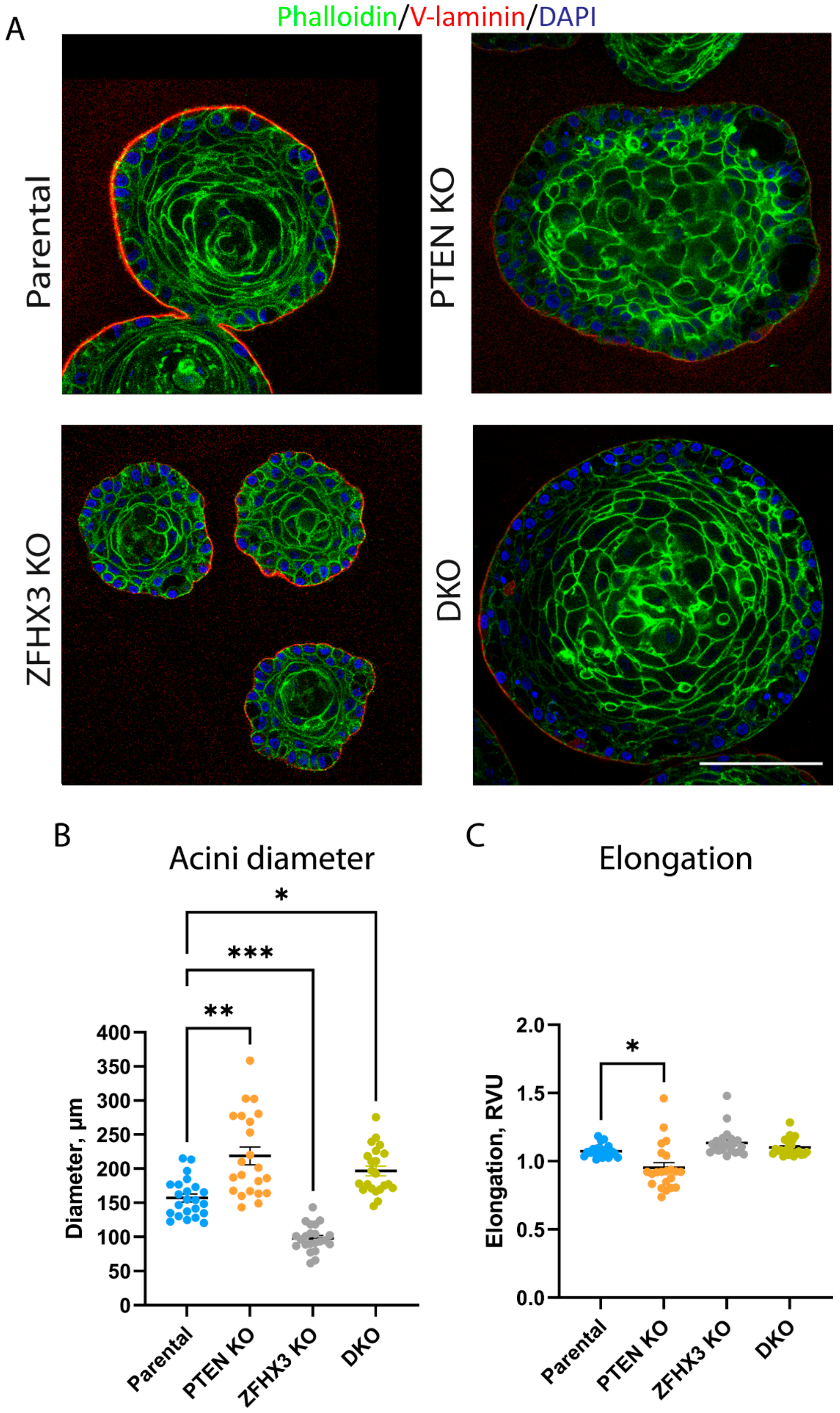

Figure S4. KOs of different TSGs induce different stages of partial EMT observed in acini morphogenesis in 3D Matrigel cell culture. (**A**) Morphogenesis of acini in 3D Matrigel. Acini were stained with DAPI (blue), phalloidin (green), V-laminin (red). (**B**) Acini diameter was measured in the center slice as a major axis. (**C**) Elongation was calculated as a ratio of major and minor axis of acini. PTEN KO or ZFHX3 PTEN DKO greatly increases the acini size, whereas ZFHX3 KO led to decrease of acini diameter (A,B). PTEN KO has an increase in invasion potential due to more elongated form of

acini. Confocal microscopy; Scale bar: 100 μm. Data are mean ± s.e.m of two technical repeats with n = 22-23. One experiment representative of two biological repeats is displayed. * $P < 0.05$, ** $P < 0.01$, *** $P < 0.001$ by Kruskal-Wallis test, non-parametric test.

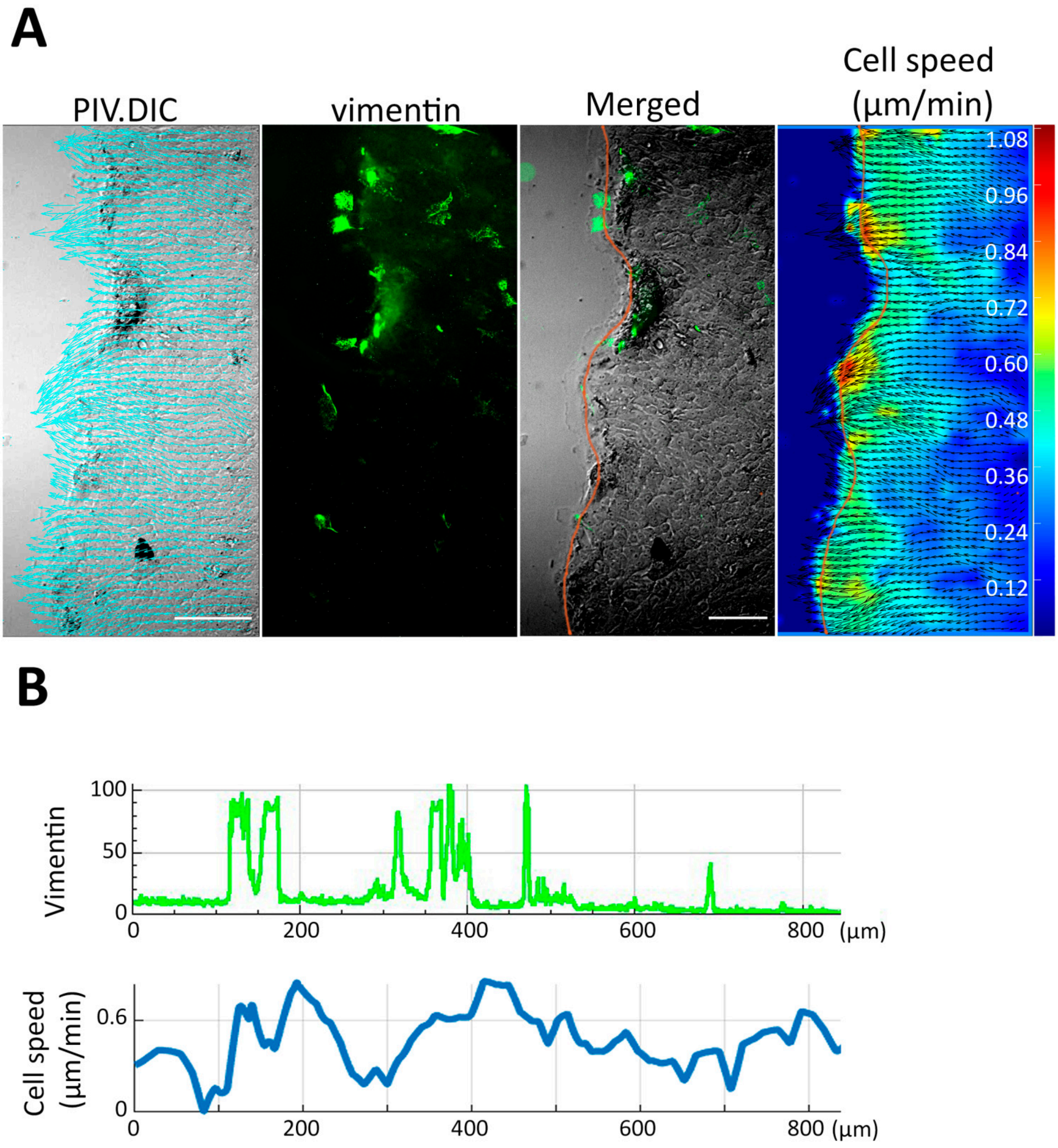

Figure S5. Fast migrating leader cells in *PTEN* KO monolayer express vimentin. (**A**) PIV analysis of *PTEN* KO monolayer upon wound healing. Cells were fixed and stained for vimentin (green) after video recording. Scale bars: 100 µm. (**B**) Cell speed and vimentin expression in cells along the edge (red line).

Table S1. Clinical characteristics of breast cancer patients and the distribution of driver genes

Case	Molecular subtype	Age	TNM	Chromosome: position	Nucleotide change	Driver genes*	Mutation type	cBioportal[‡]
KT	Lum A	41	T3N1M0	chr5: 56887461	A>AC	*MAP3K1*	frameshift insertion	7.8%
				chr3: 179234297	A>G	*PIK3CA*	nonsynonymous SNV	34.2%
				chr10: 8069550	T>TGG	*GATA3*	frameshift insertion	12.6%
MzT	Lum B HER2+	63	T2N0M1	chr3: 52403629	G>A	*BAP1*	nonsynonymous SNV	1.4%
				chr10: 8073977	C>CCTTT	*GATA3*	frameshift insertion	12.6%
				chr7: 152235864	C>A	*KMT2C*	nonsynonymous SNV	9.4%
MT	Lum B HER2-	67	T2N1M0	chr17: 16071493	C>A	*NCOR1*	nonsynonymous SNV	3.3%
				chr17: 7675127	ATGGCCATG>A	*TP53*	startloss	39.9%
				chr17: 31227595	G>T	*NF1*	stopgain	5.1%
OT	Lum A	61	T2N2M0	Not determined				
ST	Lum B HER2+	55	T1N0M0	Not determined				
ShT	Lum A	unknown	T1N0M0	chr3: 179234297	A>T	*PIK3CA*	nonsynonymous SNV	34.2%
TT	Lum B HER2+	53	T2N0M0	chr10: 87933023	T>A	*PTEN*	stopgain	5.9%
				chr16: 72957816	A>G	*ZFHX3*	nonsynonymous SNV	3.0%
XT	Lum B HER2-	59	T1N1M0	chr2: 201272745	G>C	*CASP8*	nonsynonymous SNV	0.8%
				chr7: 152163058	G>A	*KMT2C*	stopgain	9.4%
				chr11: 72018829	C>T	*NUMA1*	nonsynonymous SNV	2.3%
				chr14: 104780214	C>T	*AKT1*	nonsynonymous SNV	5.3%
YT	Lum B HER2-	38	T1N1M0	chr17: 7673764	C>T	*TP53*	nonsynonymous SNV	39.9%
SvT	Lum B HER2-	44	T2N0M0	chr6: 156778005	A>T	*ARID1B*	stopgain	3.2%
				chr19: 50413456	G>A	*POLD1*	nonsynonymous SNV	1.4%

*, according to IntOGen database (https://www.intogen.org/); [‡], mutation frequency of driver genes in breast cancer according to AACR Project GENIE; Lum, luminal; SNV, single nucleotide variation; TNM, tumor-node-metastasis classification; yr, years

Legends to supplementary movies.

Movie S1. Motility of parental MCF10A cells in depleted medium. DIC with or without tracks superimposed. Scale bar: 100 µM. Total duration 17 h 50 min.

Movie S2. Motility of *PTEN* KO cells in depleted medium. DIC with or without tracks superimposed. Scale bar: 100 µM. Total duration 17 h 50 min.

Movie S3. Motility of *ZFHX3* KO cells in depleted medium. DIC with or without tracks superimposed. Scale bar: 100 µM. Total duration 17 h 50 min.

Movie S4. Motility of DKO cells in depleted medium. DIC with or without tracks superimposed. Scale bar: 100 µM. Total duration 17 h 50 min.

Movie S5. Wound closure of genome-edited MCF10A cell lines. Scale bar: 100 µM. Total duration 7 h 50 min.
